# The Role of Postbiotics in Poultry Health and Performance: A Review on Existing Knowledge and Further Research Needs

**DOI:** 10.3390/ijms27188132

**Published:** 2026-09-12

**Authors:** Ali Zain, Moazzam Rubab, Wageha A. Awad, Dieter Liebhart, Michael Hess, Najma Arshad

**Affiliations:** 1 Institute of Molecular Biology and Biotechnology, University of Lahore, Lahore 58810, Pakistan; alisc1214@gmail.com (A.Z.); moazzamrubab1@gmail.com (M.R.); 2 Clinic for Poultry and Fish Medicine, Clinical Department for Farm Animals and Food System Science, University of Veterinary Medicine, 1210 Vienna, Austria; wageha.awad@vetmeduni.ac.at (W.A.A.); dieter.liebhart@vetmeduni.ac.at (D.L.); 3 Histovac GmbH, Magdeburggasse 10, 3400 Klosterneuburg, Austria; michael.hess@histovac.com

**Keywords:** postbiotics, poultry, antimicrobial alternatives, intestinal health, immune modulation, feed additives, growth performance

## Abstract

The widespread use of antibiotic growth promoters (AGPs) in livestock production has contributed to the emergence of antimicrobial resistance (AMR) in pathogens. Furthermore, improper dosing and insufficient withdrawal periods lead to the presence of antibiotic residues in meat, posing significant risks to human health. Consequently, regulatory bans on AGP use in poultry and livestock have increased the susceptibility of poultry to previously controlled infectious diseases, highlighting the need for effective and safe alternatives. Postbiotics, defined as preparations of inanimate microorganisms and/or their components that confer a health benefit on the host, have emerged as one of the promising AGP alternatives. Some postbiotic preparations exert immunomodulatory, antioxidant, antimicrobial, and anti-inflammatory effects, modulate gut microbiota, and reduce enteric pathogen loads, leading to improved growth performance, health status, and resilience against heat stress, thereby supporting their role in sustainable poultry production. This review summarizes the current understanding of the effects of postbiotics on poultry health and zootechnical performance and their mechanisms of action, explicitly distinguishing empirical findings demonstrated in poultry from those extrapolated from mammalian or in vitro models. Finally, it outlines knowledge gaps in the biochemical processing and standardization of postbiotics, along with future research priorities for their use as an AGP alternative.

## 1. Introduction

The continued growth of the global population has led to an increasing demand for dietary protein, underscoring the need for efficient and sustainable livestock production systems. Poultry meat is one of the most accessible and affordable sources of high-quality animal protein and contributes substantially to global food security [1,2,3,4]. Global poultry meat production exceeded 138 million tons in 2022 and is projected to grow faster than any other meat type over the coming decade, with consumption expected to increase by approximately 21% by 2034, outpacing sheep meat 16%, beef 13%, and pig meat 5%. Poultry alone is expected to provide 45% of the protein consumed from all meat sources. Much of this growth is concentrated in middle-income countries, including Pakistan, Indonesia, the Philippines, and Viet Nam [5]. Meeting this rising demand requires sustained improvements in poultry health and metabolic efficiency to support production performance. However, the productivity and sustainability of the poultry industry are increasingly threatened by various challenges such as widespread infectious disease epidemics, localized enteric pathogenic outbreaks and zoonotic pathogen transmission [3,6], the growing prevalence of antimicrobial resistance (AMR) [7], and environmental stressors that induce systemic oxidative imbalance and compromise animal performance [8].

Historically, the sub-therapeutic administration of antibiotic growth promoters (AGPs) has been widely used in poultry production for prophylactic and growth-promoting purposes [9]. However, prolonged AGP use has been associated with the emergence and dissemination of antibiotic resistance in clinically important pathogens. Furthermore, improper dosing and insufficient withdrawal periods lead to the presence of antibiotic residues in meat, posing significant risks to human health [10,11,12,13]. Consequently, regulatory restrictions and bans on AGP use have been implemented in many regions worldwide. These measures have contributed to mitigating public health risks. For instance, the banning of avoparcin as a growth promoter reduced vancomycin-resistant Enterococci (VRE) contamination in poultry meat [14]. However, the withdrawal of AGPs has exposed poultry production to numerous challenges, including production loss [15] and the re-emergence of previously controlled diseases [16]. These developments underscore the need for alternative production strategies and preventive health management approaches that can maintain poultry health and performance without reliance on antibiotics. In Pakistan, implementation of the National Action Plan on AMR (NAP-AMR) in the agriculture and food sectors remains incomplete, with persistent gaps in surveillance and regulatory enforcement in poultry production [17]. Colistin, historically used in poultry, has been linked to resistance concerns with implications for both regional and global antimicrobial stewardship [18].

Among alternative approaches, postbiotics (the preparations of inanimate microorganisms and/or their components that confer a health benefit on the host) offer a safe, highly stable, and effective alternative to AGP usage in poultry. The bioactive components in postbiotics can be broadly categorized as (i) inactivated microbial cells and cell-wall structures, including peptidoglycan and lipoteichoic acid; (ii) proteins and peptides, such as bacteriocins and enzymes, viz., proteases, lipases, amylases; (iii) nucleic acids; (iv) polysaccharides; and (v) metabolites retained within the preparation, such as short-chain fatty acids (SCFAs) and organic acids [19,20]. However, these components individually do not meet the International Scientific Association of Probiotics and Prebiotics (ISAPP) definition of a postbiotic. Purified metabolites isolated from the inactivated microbial cell or its structural components should not automatically be labeled a postbiotic. Compared to other biotic feed additives such as probiotics, postbiotics do not rely on the maintenance of microbial viability and exhibit greater stability during feed processing and throughout gastrointestinal transit [21,22,23,24]. However, inactivation does not necessarily eliminate extracellular or cellular DNA, and non-viability does not automatically ensure safety. Postbiotic preparations can retain inflammatory cell-wall structures, toxins, allergens, AMR genes, residual viable-cell contamination, or processing chemicals. Therefore, a rigorous safety evaluation through validation of the inactivation method, residual viable-cell testing, toxin screening, assessment of AMR-gene carryover, batch-to-batch consistency testing, and toxicological evaluation is mandatory for confirming the benefits of postbiotics [22,24]. The production and standardization of postbiotics requires additional downstream processing and optimization of storage conditions, as the stability of their bioactive constituents varies considerably. While metabolites such as SCFAs exhibit high thermal and storage stability, proteins and enzymes are more prone to degradation under unfavorable storage conditions [25]. Consequently, the large-scale production of standardized postbiotic preparations remains associated with significant technical and economic challenges that must be addressed to enable their widespread application in commercial poultry production systems. In spite of all these limitations, when adequately characterized, postbiotics are generally considered more predictable than live probiotics, facilitating their incorporation into feed formulations and reducing concerns associated with microbial translocation or horizontal gene transfer [26,27].

**Figure 1:**
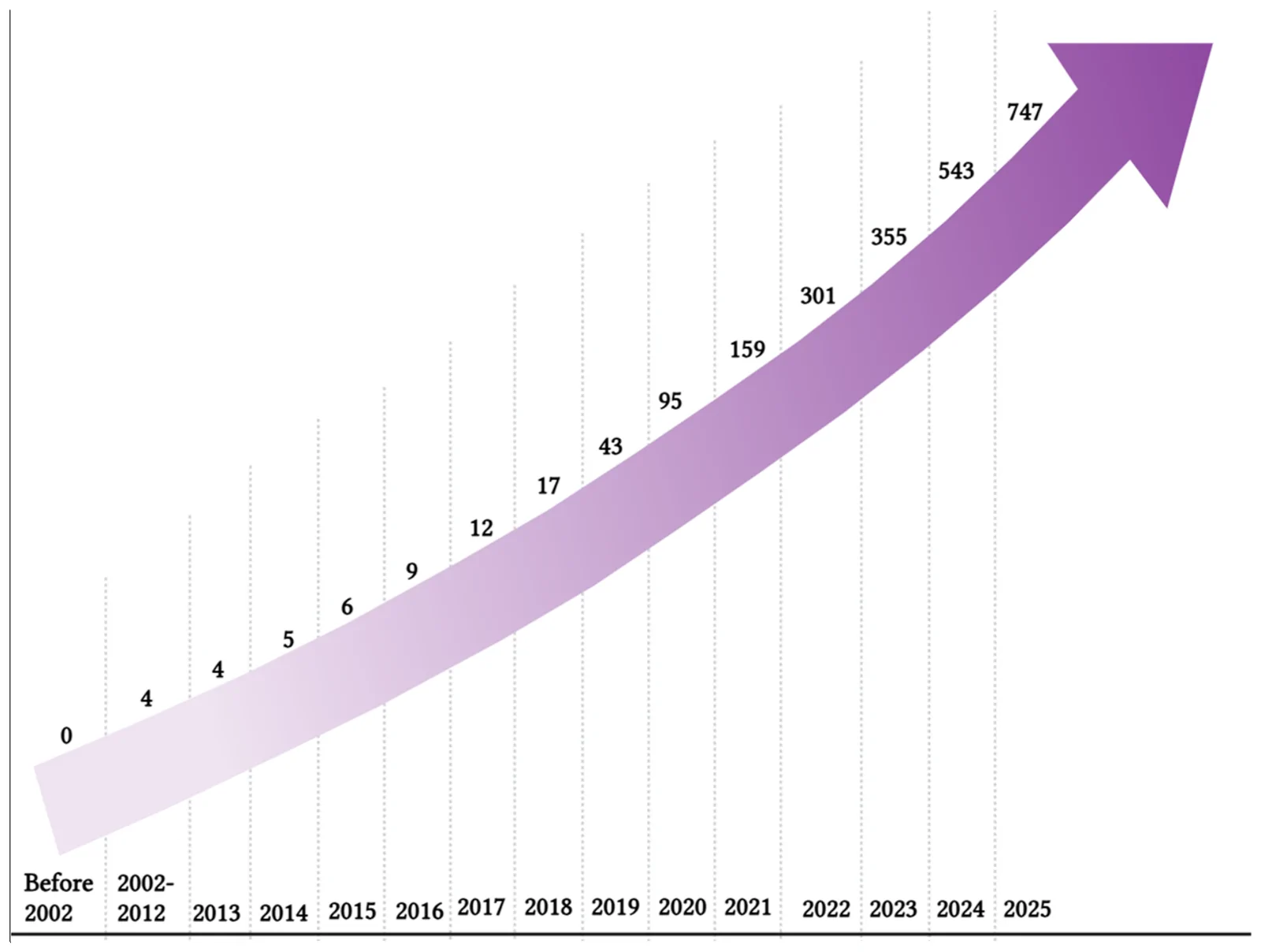
Rising trend in the use of the term “postbiotic” in published literature. Number of PubMed-indexed publications containing “postbiotic” (2002–2025); no records found before 2002. Search: PubMed, term “postbiotic” (all fields, unfiltered), Final search date: 12 August 2026.

Many terms have been coined for postbiotics including bacterial lysates: metabiotics, paraprobiotics, ghost probiotics, tyndallized probiotics, cell-free supernatant (CFS), biogenic and abiotic components, and pseudo probiotics [23,28,29,30]. These terms are not interchangeable, and only some fall within the current ISAPP postbiotic definition (Table 1). Among these classifications, the term “postbiotic” (first coined in PubMed-indexed literature in 2002) has gained increasing attention in scientific literature, with a rising number of PubMed-indexed publications (Figure 1).

Reuben et al. [33] reviewed evidence that postbiotics can improve immunity and gut health and mitigate environmental stressors, particularly heat stress. Despite these reported benefits, the practical application of postbiotics in poultry production remains a relatively novel area, constrained by limited standardization, insufficient in vivo studies, and variability in production processes.

Targeted research and clear guidelines are necessary to optimize the use of postbiotics in poultry production. This review synthesizes current evidence on postbiotic effects and proposed mechanisms of action in poultry. The specific objectives are to (i) summarize the functional roles of postbiotics in poultry health and performance and the cell-signaling pathways proposed underlying them, (ii) examine their reported roles in maintaining/protecting the intestinal barrier’s integrity against environmental and pathogenic challenges, (iii) review their potential applications in poultry production, and (iv) identify current research gaps and priorities for future investigation, including standardization and mechanistic validation in poultry.

## 2. Methodology

This is a narrative review of the literature on postbiotic use in poultry production, with the final search conducted on 12 August 2026. Literature was retrieved from PubMed and Google Scholar, covering publications from 2000 to 2025, using combinations of the search terms “postbiotic,” “paraprobiotic,” “heat-killed postbiotics,” “postbiotic AND poultry,” “postbiotic AND broiler,” “postbiotic AND layer,” and “postbiotic AND gut health”. The reproducible search string used was: (“postbiotic”[Title/Abstract] OR “paraprobiotic*”[Title/Abstract] OR “heat-killed postbiotics”[Title/Abstract]) AND (poultry[Title/Abstract] OR broiler[Title/Abstract] OR layer[Title/Abstract]). Backward and forward citation screening of included studies was also performed to identify additional relevant records. Litmaps, Research Rabbit, and Connected Papers were used as supplementary citation-mapping tools to identify additional related studies but were not treated as bibliographic databases in their own right. Due to a lack of institutional subscription access, Scopus, Web of Science, CAB Abstracts, and Embase were not searched directly; this constraint is acknowledged as a limitation in Section 9 (Challenges and Future Directions), and citation screening was used to help mitigate potential gaps in database coverage. Records were excluded if they were: (i) not published in English; (ii) conference abstracts without an associated full-text article; (iii) not conducted in poultry; (iv) in vitro only, with no in vivo/animal component; or (v) duplicate reports of the same dataset. Titles and abstracts were screened critically by the authors. Full texts of potentially eligible records were then assessed independently against the inclusion/exclusion criteria above, with disagreements resolved by the corresponding author. For each included study, data on the producer strain/product, inactivation method, dose, route, duration, comparator, and outcomes were extracted and cross-checked against the original source before inclusion in Table 2 and Table 3. Studies were included if they reported the effects of a postbiotic preparation (consistent with the ISAPP definition of inanimate microorganisms and/or their components) on poultry health, growth performance, stress response, immune function, or gut microbiota. Studies were categorized as (i) postbiotic-only interventions; (ii) postbiotic combined with prebiotic, probiotic, or other bioactive compounds, in which effects cannot be attributed to the postbiotic component alone; (iii) complex fermentation products or commercial blends whose postbiotic content was not independently verified; and (iv) preparations that did not meet the ISAPP definition. CFS were classified as category (iv) unless the primary source explicitly confirmed retention of cellular structural components following filtration. Of the 32 records meeting the inclusion criteria, 8 were classified as category (i), 1 as category (ii), 11 as category (iii), and 12 as category (iv). Findings from category (i) form the primary evidence base for this review. Studies from categories (ii)–(iv) are noted explicitly and discussed, with their compositional limitations flagged rather than presented as direct postbiotic evidence.

## 3. Definition and Types of Postbiotics

**Figure 2:**
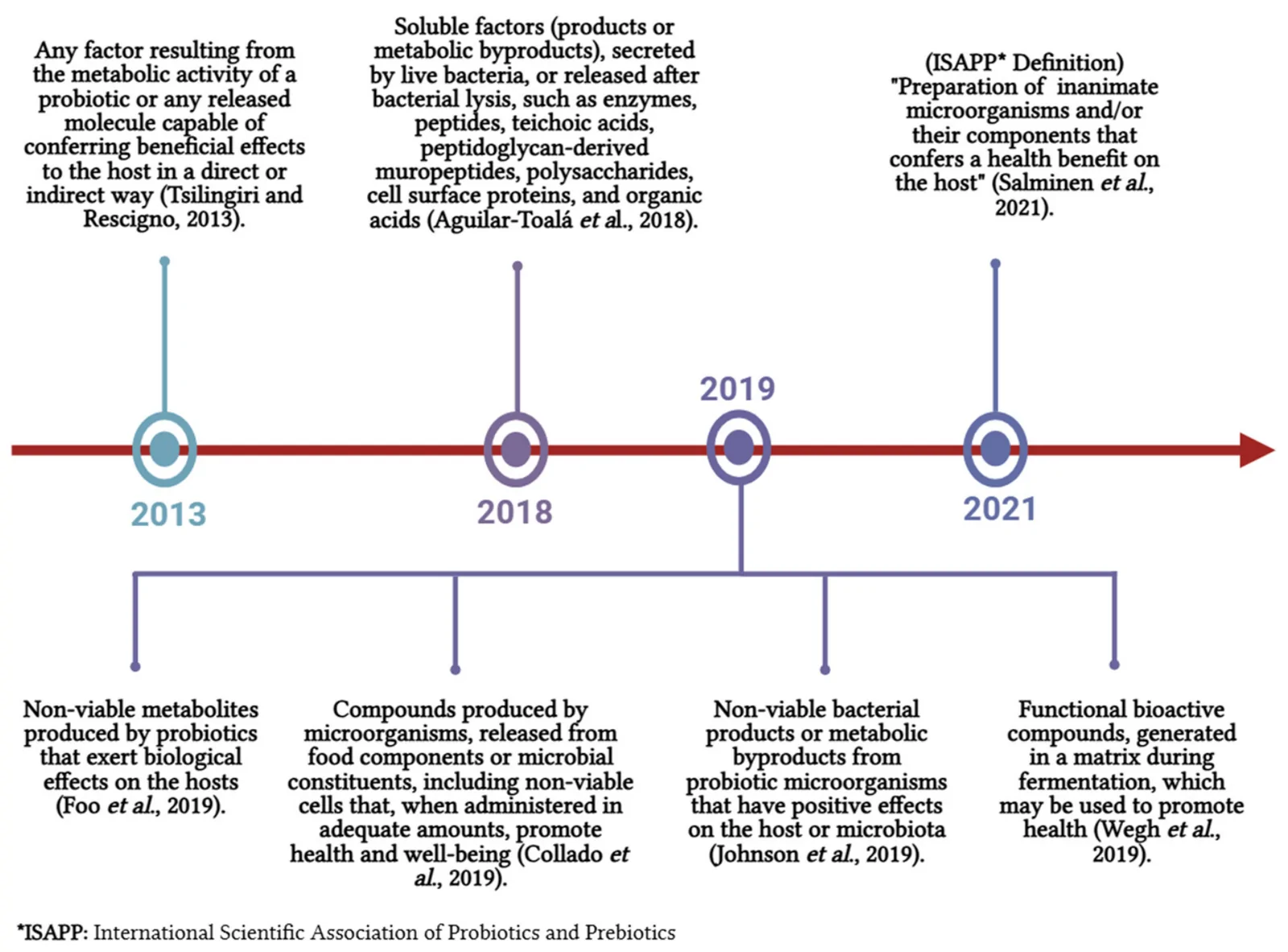
Different definitions and explanations of the term postbiotics: an evolutionary perspective. Tsilingiri and Rescigno [34], Aguilar-Toalá et al. [27], Salminen et al. [30], Foo et al. [35], Collado et al. [36], Johnson et al. [37], Wegh et al. [38].

Postbiotics represent a distinct class of bioactive preparations comprising inactivated microbial cells, cellular components, and associated metabolic byproducts. Since 2002, several definitions of postbiotics have been proposed. Some authors describe them as soluble metabolic byproducts of probiotic strains, while others include intentionally inactivated or lysed microbial cells within the concept. In May 2021, the ISAPP reviewed these varied terminologies focusing on metabolite bio-products of probiotics, cell wall fragments, and extracellular components. The panel issued one statement of a consensus to define postbiotics which has become a widely cited scientific reference point, though terminology and regulatory recognition still vary internationally. According to the ISAPP consensus, a postbiotic is defined as “a preparation of inanimate microorganisms and/or their components that confers a health benefit on the host” [30]. The chemically purified metabolic end products derived from producer strains, in the absence of retained cell biomass or structural components, are generally excluded from this definition. Historical perceptions of the term “postbiotics” are presented in Figure 2. Postbiotics can be derived from both bacterial and fungal/yeast sources.

### 3.1. Bacterial Sources of Postbiotics

Bacterial postbiotics are primarily derived from lactic acid bacteria (LAB) within the phylum *Bacillota*, including *Lactiplantibacillus plantarum* (*L. plantarum*), *Lactobacillus acidophilus*, *Lactobacillus delbrueckii*, *Lactobacillus helveticus*, *Lactobacillus johnsonii*, *Limosilactobacillus reuteri*, *Lacticaseibacillus rhamnosus*, *Lacticaseibacillus casei*, *Lacticaseibacillus paracasei*, *Limosilactobacillus fermentum*, and *Lactobacillus gasseri*, as well as *Bifidobacterium* species (phylum Actinomycetota). Some strains of *Bacillus subtilis*, having probiotic properties, are also used for postbiotic production. LAB species have been reported as a source of postbiotics with variable effects on gut health modulation, immune system, and antioxidant status, depending on strain, dose, and preparation method [39,40,41,42,43,44,45,46]. Among these, strains of *L. plantarum* are the most commonly studied postbiotic producers, reported to activate host signal transduction networks [33,47,48]. These effects of postbiotics derived from LAB species have also been investigated in broilers and layers [44,49,50,51,52,53]. Their improvements in meat quality and redox potential under heat-stress conditions have also been reported by some authors [51,54].

### 3.2. Yeast/Fungal Sources of Postbiotics

The yeast-derived postbiotics are obtained mainly from *Saccharomyces cerevisiae* (*S. cerevisiae*). Among these, *S. cerevisiae* fermentation products (SCFP) have been characterized as containing bioactive compounds such as organic acids, peptides, nucleotides, polyphenols, and cell wall components. Whether a given SCFP consists of inactivated yeast cells and components, retained metabolites, residual viable organisms, or unfermented substrate material should be specified case by case, as this determines whether it meets the ISAPP postbiotic definition. SCFP has been reported to support gut health, immunity, and production performance measures in commercial poultry. The supplementation of SCFP reduces intestinal lesions and improves immune functions during coccidial (*Eimeria*) challenge, improves productivity [55] and immune functions [56], improves the feed conversion ratio (FCR) [57], meat yield, and egg production [58], and possibly decreases colonization by *Salmonella enterica* serovar Enteritidis (SE) [59,60]. SCFP has also been reported to lower corticosterone levels, maintain the heterophil-to-lymphocyte ratio during stress events, and reduce physical asymmetry [58]. Physical asymmetry is a measure of bilateral variation in paired body traits such as metatarsal length, which is used as an indicator of developmental and physiological stress.

## 4. Composition of Postbiotics

**Figure 3:**
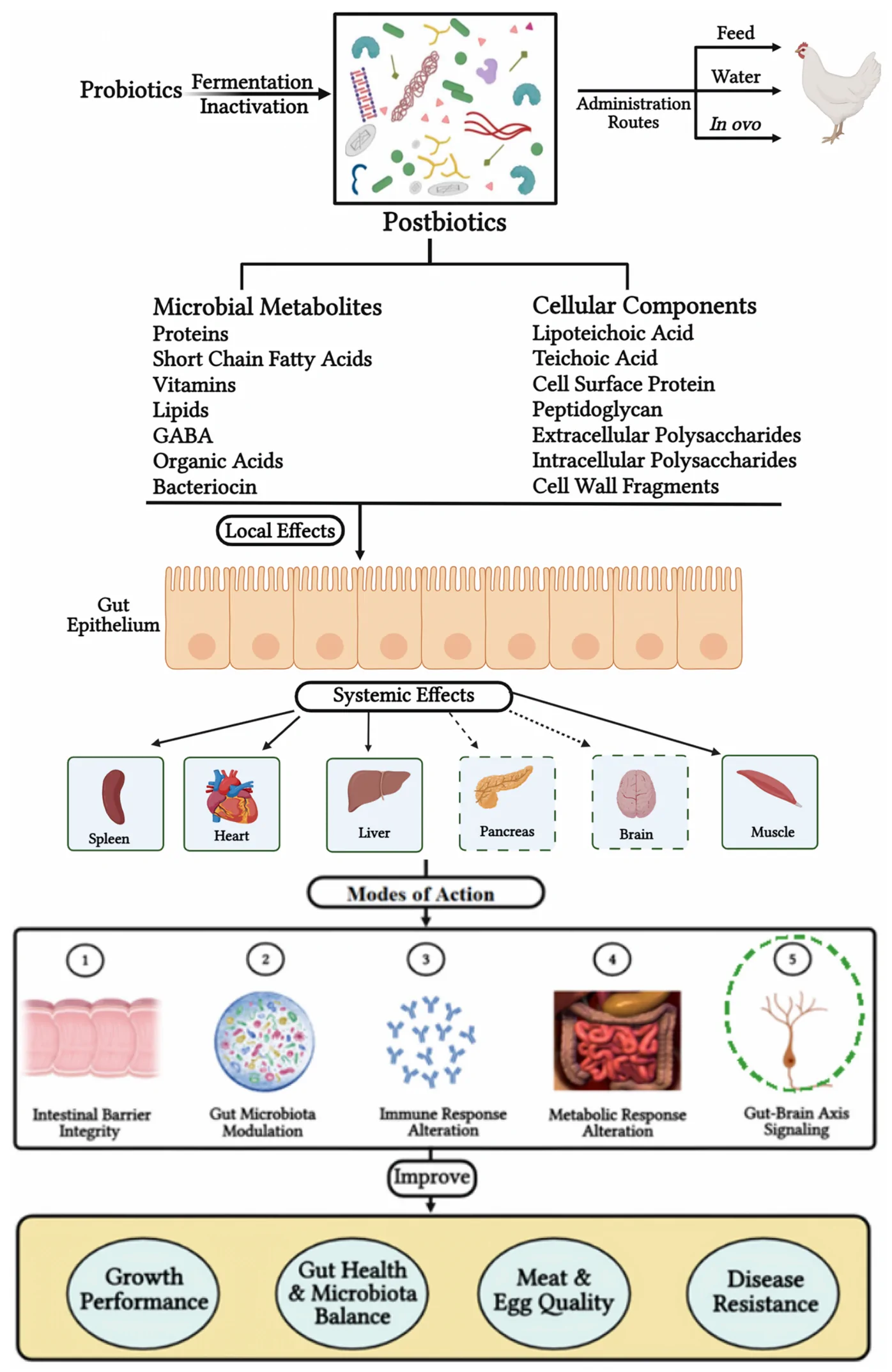
Components, administration routes, systemic distribution, modes of action, and final outcomes of postbiotics in poultry. Solid arrows and borders indicate effects directly demonstrated in poultry; dashed arrows and borders indicate effects extrapolated from evidence in other species and considered hypothetical in poultry. Information on gut [42], gut barrier integrity [61], spleen [40], heart [62], muscle-related antioxidant [63] and liver [40] is based on literature in poultry. Effects on pancreas, brain and gut–brain axis signaling are extrapolated from literature in other species [64,65] and are hypothetical in poultry.

A comprehensive understanding of the potential benefits of postbiotics in poultry requires an exploration of the specific roles of their individual components, which can be broadly divided into two categories: (i) cellular components and (ii) microbial metabolites/byproducts (Figure 3).

Some of these components are absorbed across the intestinal epithelium and may exert effects beyond the gut, while others act locally through direct interaction with gut epithelial receptors. In poultry, local effects on gut health and nutrient digestibility are relatively well documented [42,66]. Among distal-organ effects, direct evidence in poultry exists for the liver and skeletal muscle. Postbiotics have been shown to regulate hepatic lipogenesis via a GLP-1-dependent mechanism in broiler chickens [67], and SCFA/protoporphyrin IX metabolites have been shown to regulate skeletal muscle fiber size and glucose metabolism in chickens [68]. Cecal SCFA levels have also been correlated with abdominal adipose tissue development in broilers, though this association has not yet been established through a direct postbiotic feeding intervention [69]. By contrast, proposed effects on the pancreas and brain/behavioral outcomes including “mood regulation”, remain hypothetical in poultry: the underlying mechanisms are drawn from mammalian and human SCFA research [70] and have not, to date, been directly demonstrated in birds.

Consistent with the ISAPP definition, postbiotic composition is best organized into three categories that reflect whether cell biomass is retained in the final preparation. The first category comprises inanimate microbial biomass and structural components. This matrix consists of distinct cellular fractions including teichoic acid (TA), lipoteichoic acid (LTA), cell surface proteins, peptidoglycans, extracellular polysaccharides (EPS), cell wall fragments and intracellular polysaccharides [23]. The second category involves metabolites retained within the preparation alongside this biomass. These metabolites include proteins (p75, p40 molecules), enzymes (proteases, lipases, and amylases), SCFAs (acetate, propionate, and butyrate), vitamins, lipids, gamma-aminobutyric acid (GABA), lactic acid, 3-phenyl-lactic acid, and bacteriocins [26,32]. The third category addresses purified microbial isolates evaluated in the absence of retained cell biomass (such as GABA, SCFAs, vitamins, or bacteriocins studied alone). Preparations in this third category do not, by themselves, constitute a postbiotic under the ISAPP definition and are therefore classified separately from postbiotic preparations. Fiaz et al. [71] identified a list of ethyl acetate-soluble metabolites in CFS of *L. plantarum*-ZFS (PP832863) using in vitro and computational (machine-learning/bioinformatics) methods. These components of postbiotic preparations have been reported to exert local effects on gut epithelium, including anti-inflammatory, immunomodulatory, and antimicrobial activity. Reported systemic effects, including immunomodulatory and antioxidant activity, have some direct support in poultry [72], whereas anti-hypertensive and anti-proliferative effects have been reported primarily in mammalian or in vitro systems and should be regarded as extrapolated rather than established in poultry.

## 5. Modes of Action

The beneficial impacts of postbiotics in poultry are facilitated by a variety of complex and interrelated modes of action. These effects could be summarized in five key areas: (1) reinforcement of intestinal barrier function, (2) modulation of the gut microbiome, (3) immune system regulation, (4) metabolic response alteration, and (5) gut–brain axis signaling (Figure 3).

### 5.1. Intestinal Barrier Function and Gut Microbiome Modulation

Postbiotics have been reported to support intestinal barrier integrity and gut microbiome balance in poultry through several interconnected mechanisms [42,54]. The intestinal epithelial barrier constitutes the primary line of defense against enteric pathogens and dietary toxins, comprising a single layer of epithelial cells sealed by tight junction complexes that regulate paracellular permeability and protect the host from luminal antigens. Disruption of this barrier increases intestinal permeability, permits translocation of gut microbiota across the epithelium, and triggers systemic inflammation [73]. In broiler chickens, Awad et al. [73] demonstrated that enteric pathogens including *Campylobacter jejuni*, SE, and *Clostridium perfringens* (*C*. *perfringens*) disrupt tight junction integrity through toxin-mediated mechanisms. The mycotoxin deoxynivalenol (DON), a common poultry feed contaminant, reduces the expression of mucins and tight junction proteins, disrupts nutrient absorption, and facilitates the translocation of *Escherichia coli* (*E. coli*) across the gut wall [74,75,76]. Postbiotic supplementation has been reported to upregulate the expression of tight junction and mucin genes, including occludin, claudin-1, zonula occludens-1 (ZO-1), and MUC2, in both broilers and layers [42,54,77,78], supporting a direct barrier-reinforcing role in poultry. The pathogens and dietary contaminants that compromise intestinal barrier integrity can severely affect health, particularly meat production outcomes (Table 2).

**Table 2:** Overview on studies investigating the effects of postbiotics administered via feed and/or water in chickens.

Sr. No.	Producer Strain/Product	ISAPP Cat	Inactivation Method	Dose	Route	Duration	Bird Type	Sample Size	Comparator	Key Stat Results	Ref
1	*L. plantarum* postbiotics	(i)	Heat-killed	320 mg/kg	Feed (mixed into basal diet)	42-day feeding trial (day-old to market age)	Broilers	*n* = 480 (4 groups × 6 replicates × 20 birds/replicate)	Control; probiotic-only *B. subtilis*; postbiotic-only heat-killed *L. plantarum*	Increased dressing-out %, spleen/bursa of Fabricius indices; growth performance improvements	[79]
2	SCFP	(iii)	Fermentation-derived	Standard commercial inclusion (dietary)	Feed	3 rearing cycles, ceca sampled at ~33–34 d	Broilers, commercial farm (Honduras)	12 houses (6 CON/6 SCFP), 25 ceca sampled per house	Standard industry diet (CON)	Cecal *Salmonella* prevalence: 12.2% (CON) → 3.4% (SCFP), *Salmonella* load in positive litter samples also reduced	[59]
3	SCFP	(iii)	Fermentation-derived	1.25 kg/metric ton feed	Feed	0–42 days	Broilers, Cobb 500	300 birds/treatment (4 × ~75/trial, 3 trials)	Nonstressed control; stressed control	Reduced physiological stress markers improved bilateral bone-trait symmetry	[80]
4	Stabilized non-viable *Lactobacilli*, aqueous form (Postbiotic)	(i)	Stabilization process (non-viable cells)	4 mL/L drinking water	Water-administered (one arm of a 4-arm feed/water comparison study)	35 days total trial	Broilers, day-old, *n* = 400 total	*n* = 400 (4 groups × 100 birds, 2 replicates of 50 each)	G1: dry form; G2: aqueous form; G3: both dry + aqueous; G4 challenged control	Aqueous arm: reduced *E. coli*-related lesions, improved antibody titers	[22]
5	*L. plantarum* + SCFP coconut water	(iv)	Fermented coconut water (*L. plantarum* MS2c + *S. cerevisiae* Y301)	0.16–0.3% (MY5/M2 groups)	Water	21 days total (7-day DSS induction + 14-day recovery)	Wenchang chicken (a Chinese native breed)	*n* = 60 14-day-old chick DSS-enteritis model	DSS-induced enteritis model vs. non-challenged/treated comparison	Improved growth by increasing feed intake and body weight in chicks recovering from DSS-induced acute enteritis	[81]
6	Postbiotic (dry feed additive + aqueous non-viable *Lactobacillus* fermentation)	(i)	Non-viable fermentation product	Dry: 1 kg/t starter, 500 g/t grower + finisher; Aqueous: 4 mL/L water	Feed and Water (3-arm: dry only, water + antibiotic, dry + aqueous) vs. probiotic and antibiotic arms	NE challenge d19–21; total 35 d	Broilers, day-old	*n* = 400 (8 groups × 50, 5 replicates of 10 each)	Probiotic (*B. subtilis* + *B. licheniformis*, 200 g/t) + Antibiotic (Amoxicillin 20 mg/kg BW)	Highest NDV antibody titers in postbiotic groups (dry and dry + aqueous arms)	[82]
7	Postbiotic (non-viable bacterial product/metabolic byproduct)	(i)	Non-viable bacterial postbiotic	1 oz/gallon drinking water	Drinking water—1 oz/gallon, added to water jugs/buckets from day 1	*C. perfringens* challenge model; jejunal tissue kinome analysis	Broilers	21 d (2 replicate trials)	Challenge vs. no challenge, with/without postbiotic	Improved weight gain and lesion scores, reduced *C. perfringens* counts and mortality	[37]
8	Postbiotic metabolite combinations, *L. plantarum* strains	(iv)	Cell-free liquid metabolite (fermentation supernatant)	0.6% metabolite combinations (*v*/*w*) in feed	Feed	12 weeks (23–35 wk of age) + 1 wk adaptation	Laying hens	200 Lohman Brown hens, 4 groups, 25 replicates × 2 birds/cage	Control vs. 3 metabolite blends	Reduced plasma/yolk cholesterol; enhanced egg quality and antioxidant capacity; reduced lipid oxidation	[51]
9	*L. plantarum* RI11 postbiotic (CFS)	(iv)	Fermentation-derived CFS, not whole-cell 0.22 µm filtered (postbiotic RI11)	0.2%, 0.4%, 0.6%, 0.8% (*v*/*w*)—4 dose levels	Feed	d22–42 (20 d treatment; 42 d total), cyclic heat stress 36 ± 1 °C, 3 h/day	Male broilers, Cobb 500	*n* = 252 (7 treatment groups)	Negative control (0%); positive control (0.02% oxytetracycline); antioxidant control	0.6% optimal: ↑ plasma GPx/catalase/glutathione; ↑ ZO-1/MUC2/CLDN1/OCLN mRNA; ↓ HSP70, cytokine	[54]
10	*L. plantarum* postbiotics (strains RG11, RI11, RS5)	(iv)	CFS centrifuged + 0.22 µm filtered	0.1% (*v*/*w*) each strain (RG11/RI11/RS5)	Feed	42 d (sampled d21 & d42)	Male Cobb 500 day-old chicks	*n* = 245 (5 treatments × 7 replicates × 7 birds)	CRD design; Neg Control, OTC (0.01% *w*/*w* oxytetracycline)	↑ GSH-Px, SOD antioxidant activity; ↓ intestinal MDA (lipid peroxidation)	[42]
11	SCFP	(iii)	SCFP metabolites (Original XPC)	1.5 kg/MT (d0–21) → 1.0 kg/MT (d22–32)	Feed (preharvest)	32 d; SE challenge (1 × 10^6^ CFU) d28	Layer pullets	66 Hy-Line W-36 (NCON = 20, PCON = 20, SCFP = 19)	NCON (no challenge), PCON (challenge, no additive)	Reduced SE in ceca as a preharvest food-safety hurdle	[58]
12	*L. plantarum*	(iv)	Postbiotic = CFS; paraprobiotic = heat-inactivated cells	0.2% (*v*/*w*) in feed, 6 strains/forms	Feed	35 d (d0–21, d22–35)	Broilers, Cobb 500, day-old	*n* = 336 (8 treatments × 6 replicates × 7 birds)	NC, PC (0.01% *w*/*w* oxytetracycline)	Effects on colon mucosa microbiota composition	[83]
13	*L. plantarum* postbiotic	(iv)	CFS (LPC), not heat-killed	0.8% in drinking water	Water	28 d pretreatment, then SE challenge (1 × 10^9^ CFU)	Broilers	240 day-old male yellow-feathered broilers	Control, SE-only, LPC, LPC + SE (4 groups)	Increased microbial diversity; reduced *Salmonella* infection rate, gut microbiota changes	[84]
14	*L. plantarum* and its fermentation products	(iii)	Live *L. plantarum* vs. its fermentation product	0.1%/0.15% (live) vs. 3%/4% (ferment. product)	Feed	42 d	Broilers	1200 Lingnan yellow broilers, 5 groups, 6 × 40	CK control vs. live bacteria vs. fermentation product doses	Growth performance, immune function, intestinal pH, cecal effects	[85]
15	*L. plantarum*-derived postbiotic	(iv)	CFS	0.3%	Feed	35 d (d1–18, d19–35)	300 Ross 300, 5 groups, 3 × 20 Broilers	NC, PC Probiotic Prebiotic	Probiotic arm; prebiotic arm; postbiotic arm	Improved meat tenderness and water-holding capacity in postbiotic groups	[62]
16	*L. rhamnosus* CNCM I-3698 + *L. formosensis* CNCM I-3699	(i)	Heat-inactivated	METALAC: 500 g biomass/t	Feed	35 d (sampled d21, d35)	Broilers	1368 male Ross 308	Diet composition × additive factorial design	Improvements in growth and physiology	[86]
17	Yeast cell wall extract vs. postbiotic yeast cell wall blend	(iii)	YCWE vs. PYCW (postbiotic yeast cell wall blend)	0.2% each	Feed	42 days, with interim sampling at day 21	Broilers	720 male Cobb, 6 groups	Mycotoxin challenge vs. mitigation groups	Mitigation of natural fusarial multi-mycotoxin challenge effects by postbiotics.	[87]
18	*S. cerevisiae*-derivatives	(iii)	Fermentation	250 g/t feed	Feed and water	5 d florfenicol exposure within broader trial	Broilers	600 Cobb-500, 4 groups × 3 × 50	G3 florfenicol (25 mg/kg BW, antibiotic), G4 combo	Growth, antioxidant capacity, immune response improved	[77]
19	SCFP	(iii)	SCFP (Diamond V Original XPC)	1.25 kg/MT	Feed	42 d	Vencobb 400 Broiler	324 Vencobb 400, 3 groups, 12 × 9	T1 control, T2 probiotic, T3 SCFP (postbiotic)	Efficacy of SCFP in weight gain and improved FCR	[57]
20	*S. cerevisiae*	(iii)	Distiller’s dried yeast	3% (starter) → 6% (grower) → 9% (finisher)	Feed	Starter–grower–finisher to 37 days	Broilers	112 chicks, 28 boxes × 4	Control vs. yeast-supplemented	Effects on selected microbiotic fractions, carcass and meat quality	[88]
21	SCFP	(ii)	Fermentation	Prototype dose (Dia-V™ PTPLUS, Diamond V)	Feed	APEC O78 intratracheal challenge at d28; necropsy d35 and d42	Broilers	*n* = 600 (5 treatment groups)	Challenged control; vaccine alone; SCFP alone; SCFP with vaccine	Treatments improved lesion scores and reduced blood cytokines	[89]
22	Strains of *L. reuteri*/*L. plantarum*	(i)	3-way: viable heat-inactivated CFS of same *Lactobacillus* mix	~2.2 × 10^9^ CFU/mL, 1 mL/bird	Oral gavage	35 days	Broilers	252 broilers, 7 groups	Control, DON, CP, CP + DON, VL, HIL, LCS—explicit probiotic/paraprobiotic/postbiotic design	Modulation of broiler intestinal changes induced by *C. perfringens* and DON	[90]
23	*L. plantarum* postbiotics	(iv)	CFS, (10,000× *g*/15 min)	300 mL solution per 100 kg feed	Feed	Factorial 2 × 3 design	Layer hens	192 layer hens, 6 groups × 32/rep	Heat stress × postbiotic factorial	Performance effects under heat stress in layer hens	[91]
24	*Bacillus subtilis* ACCC 11025	(i)	Heat-killed/inactivated (postbiotic)	0.000, 0.015, 0.030, 0.045% (4 dose levels)	Feed	42-day study, day-old to market age	Arbor Acre broilers (day-old)	*n* = 480 (4 groups × 6 replicate cages × 20 birds)	Basal diet (0% postbiotic) as negative control; dose–response comparison across 4 levels	0.015% optimal: ↑ growth performance, ↑ AIV/NDV antibody titers, ↑ serum albumin/total protein, ↓ mortality rate	[92]
25	Concentrated SCFP	(iii)	Fermentation-derived	0.625 kg/MT	Feed	d42; feed withdrawal/heat stress d18	Broilers	Day-old Cobb 500 males, 2 group, 15 reps	Control vs. treated	Improved FCR and reduced physiological stress markers	[93]
26	Inactivated whole-cell *Pichia guilliermondii* (yeast product)	(i)	inactivated whole-cell yeast postbiotic	0.5 kg/t	Feed	40–46 wk of age (6 wk)	Layers (egg production)	35 hens, 5 groups, *n* = 7/treatment	Control vs. 4 yeast products	Preliminary assessment of dietary yeast products on egg production	[94]
27	*S. cerevisiae* metabolite	(iii)	Fermentation-derived	1000 ppm	Feed	Wk 26–37 of age (breeder production period)	Broiler Breeder Hen	*n* = 240 (120 wet-litter + 120 high-performing)	Between-line comparison; SC-supplemented vs. control within each line	Improved egg production/hatchability and reduced mortality	[95]

Therefore, postbiotics have been investigated for their ability to improve intestinal barrier integrity and gut health. Mechanistically, postbiotic cell-wall components such as lipoteichoic acid and peptidoglycan are recognized by host pattern recognition receptors, a process well characterized in chickens; avian Toll-like receptor 2 recognizes bacterial lipoproteins and peptidoglycan in a manner functionally analogous to its mammalian counterpart [96], while TLR4-mediated recognition of bacterial ligands activates heterophils and upregulates pro-inflammatory cytokine and chemokine expression [97]. Bacterial quorum-quenching activity has similarly been proposed as a route by which postbiotic-associated compounds interfere with pathogen virulence and biofilm formation [98]. In the human-associated probiotic strain *Lactobacillus rhamnosus* GG, surface fimbriae and lectin-like molecules competitively bind pathogen adhesion sites and suppress epithelial colonization in vitro [99,100]. Whether an analogous mechanism operates for postbiotic preparations in the avian gut remains to be tested directly. Cross-feeding between postbiotic-derived organic acids and resident SCFA or bacteriocin-producing taxa has similarly been characterized in human colonic bacterial co-cultures [101], offering a plausible model for postbiotic-microbiota interactions in poultry, though bacteriocin production itself is strain-specific rather than a general consequence of organic-acid availability. Collectively, postbiotic-associated barrier-protective effects against enteric pathogens [42,61,102] and DON [87], together with direct induction of tight-junction gene expression, are supported by multiple avian studies. Although the underlying receptor- and microbiota-level mechanisms are increasingly supported by avian-specific data, the exact biological pathways and full mechanistic network have not yet been completely explored. Reported effects are not universal, however. Forder et al. [95] found that dietary inclusion of *S. cerevisiae* metabolite improved reproductive performance in two chicken meat breeder lines but did not affect intestinal permeability, indicating that postbiotic benefits on barrier function are not observed uniformly across all study designs and endpoints.

### 5.2. Immune System Regulation

Postbiotics modulate both local and systemic immune responses in poultry through interactions with host immune cells. As mentioned above, the microbe-associated molecular patterns (MAMPs) such as lipoteichoic acid (LTA) and peptidoglycan are recognized by pattern recognition receptors (PRRs) including Toll-like receptors (TLRs) 2 and 4 and NOD-like receptors (expressed on chicken immune and epithelial cells) [96,97,103]. This receptor-level recognition provides a plausible avian mechanistic basis for the barrier- and immune-modulating effects of postbiotic cell-wall fragments, complementing broader descriptions of MAMP-driven NF-κB signaling in the wider probiotic literature [103,104]. Depending on MAMP structure, the receptor engaged, and tissue context, this recognition can enhance anti-inflammatory signaling through the production of IL-10 or promote pro-inflammatory cytokine and chemokine expression via NF-κB activation [105]. The direct evidence for postbiotic-mediated immune modulation in poultry is substantial: Postbiotic supplementation has been associated with reduced pro-inflammatory cytokine expression and improved survival during colibacillosis [89], increased antibody titers and circulating leukocyte counts [77], reduced mortality linked to attenuated intestinal dysbiosis, upregulated IL-10 alongside reduced pro-inflammatory markers [54], and enhanced secretory IgA response [42]. These findings are summarized in Table 2. Postbiotic administration has also been shown to stimulate the phagocytic activity of macrophages and heterophils in broiler chickens, thus bolstering the host’s defense against infection [37]. The secreted bacterial proteins p75 and p40 have additionally been reported to enhance mucosal immune defenses by activating the epidermal growth factor receptor (EGFR) pathway in intestinal epithelial cells (IECs), reinforcing barrier function and promoting anti-inflammatory signaling [32]. This specific mechanism has been characterized primarily in human intestinal cell models and represents a promising target for validation in avian epithelium.

### 5.3. Systemic Metabolic Control

Beyond local gut effects, postbiotics have been associated directly and indirectly with systemic metabolic outcomes in poultry, including nutrient utilization, altered lipid metabolism, and energy balance. Postbiotics help increase the growth of beneficial gut microbes which produce SCFAs and secondary bile acids. These components interact with host receptors and regulate metabolic pathways involved in energy, lipid, and glucose homeostasis. In mammalian systems, secondary bile acids generated through the resident gut microbiota interact with host nuclear and membrane receptors (FXR and TGR5) to regulate metabolic genes controlling lipid, glucose, and energy balance [106]. This mechanism has not yet been directly confirmed in poultry and is proposed here by analogy. If established, an equivalent pathway could plausibly contribute to decreased intestinal inflammation, improved nutrient absorption, and better feed efficiency in poultry.

Postbiotic-associated metabolic effects most plausibly arise through modulation of this resident microbiota-bile-acid axis rather than direct bile-acid transformation by the postbiotic preparation itself. SCFA-induced GLP-1 secretion has been shown to link gut microbiome composition to hepatic lipogenesis in broiler chickens [67]. GLP-1 has separately been confirmed to increase satiety and inhibit feed intake in chickens, while intracerebroventricular GLP-1 administration reduces blood glucose level and affects energy metabolism [107,108]. These effects are further supported by the expression of GLP-1 receptors expressed in broiler intestinal and hepatic tissue [109]. These findings indicate that gut-derived metabolites can plausibly link postbiotic supplementation to systemic energy and appetite regulation through a genuinely avian-confirmed GLP-1 pathway. The specific mechanism by which GLP-1 stimulates pancreatic β-cell insulin secretion and delays gastric emptying, as characterized in mammalian pharmacology, has not yet been directly tested in poultry and should be regarded as inferred by analogy pending direct avian confirmation. Consistent with the confirmed avian finding, postbiotic supplementation has been associated with reduced plasma and yolk cholesterol and enhanced beneficial fatty acid content in laying hens [51], along with reduced circulating blood lipids in broilers [77] (Table 2). Postbiotics derived from *L. plantarum*, *L. acidophilus*, and *L. casei* have also been reported to retain B-group vitamins and lipid metabolites that contribute to improved health and performance [26]. Together, postbiotic bioactive compounds support recovery after infection, optimize energy and nutrient utilization, and reduce metabolic stress [110], though this composition-function relationship has been characterized primarily outside poultry systems and warrants direct confirmation.

### 5.4. Gut–Brain Axis

An emerging body of evidence from non-avian studies suggests postbiotics may influence the gut–brain axis in poultry through the modulation of neural signaling, neurotransmitter release, and stress-related physiological pathways. This remains to be established and is currently the least-characterized mechanism in avian systems. The gut–brain axis is a bidirectional communication system linking the gut microbiota and the central nervous system (CNS). In mammalian systems, SCFAs have been shown to stimulate serotonin release from intestinal enterochromaffin cells, with serotonin acting as a neuromodulator that influences gut motility, appetite, and stress-related behavior [111]. Whether postbiotic-derived SCFAs elicit an equivalent serotonergic response in poultry has not yet been directly investigated. In addition, certain components of postbiotics such as GABA are bioactive and can modulate the gut–brain axis through enteric neural and vagal signaling pathways. In broilers, postbiotic supplementation has been associated with reduced circulating corticosterone and a lower heterophil-to-lymphocyte ratio [93] (Table 2). The established physiological indicators of the avian stress response provide indirect but measurable support for a stress-attenuating effect consistent with gut–brain axis modulation in mammals. Direct characterization of the neural signaling components of this pathway in poultry remains an important target for future investigation.

## 6. Functional Impacts of Postbiotics in Poultry

### 6.1. Growth Performance and Feed Efficiency

Postbiotic supplementations have been associated with improved growth performance and feed efficiency in poultry, generally linked with enhanced gut health, antimicrobial activity, and improved nutrient utilization (Table 2). They also play a role in weight gain, which translates to enhanced growth performance in poultry (Figure 3). Girija et al. [72] reported that *L. plantarum*-derived postbiotics improved body weight and lowered FCR in broilers; comparable findings were reported by Kareem et al. [112]. Humam et al. [43] and Farran et al. [91] reported that postbiotics improved nutrient absorption as well as gut health, which further contributed to increased feed efficiency and weight gain in heat-stressed birds. Under heat-stress conditions, Humam et al. [43] further reported that different *L. plantarum*-derived postbiotics improved growth performance, carcass yield, intestinal morphology, gut microbiota composition, and growth hormone receptor/IGF-1 gene expression in broilers exposed to cyclic heat stress. Reported effects are not uniformly positive across all endpoints, however. Heinsohn et al. [93] found that a concentrated SCFP product lowered FCR and reduced physiological stress markers in heat-challenged broilers, but did not report a statistically significant difference in final body weight, illustrating that postbiotic effects can be endpoint-specific rather than uniformly beneficial across all growth parameters.

### 6.2. Gut Health and Pathogen Resistance

Growing scientific interest in postbiotics reflects their reported contribution to gut integrity and pathogen resistance in poultry [83]. Postbiotics have been associated with favorable shifts in gut microbiota composition, limited pathogen colonization, and maintained intestinal barrier integrity. In a related line of evidence, live supplementation of *L. plantarum* has been shown to relieve signs of necrotic enteritis (NE) associated with *C. perfringens* colonization in birds [113], illustrating the broader probiotic/postbiotic functional space from which postbiotic mechanisms are often inferred. Working with a genuine *L. plantarum*-derived postbiotic, Guan et al. [84] reported increased microbial diversity and a reduced *Salmonella* infection rate in poultry. The authors attributed these findings to modulation of gut microbiota composition and NLRP3 inflammasome-related immune signaling rather than classical live-organism competitive exclusion. Collectively, postbiotics have been associated with reduced incidence of enteric disease, a primary challenge in poultry production, making them relevant for sustainable and antibiotic-free poultry farming.

Not all outcomes are uniformly positive, however. Danladi et al. [83] reported an increase rather than a decrease in cecal Proteobacteria abundance with paraprobiotic strain RG14. In Guan et al. [84], pre-challenge growth performance, immune organ indices, most cecal volatile fatty acid concentrations, and microbial alpha diversity were not significantly affected and the suppression of TLR4/MyD88/NF-κB signaling was significant in the jejunum but not the ileum. de Souza et al. [90] similarly found that postbiotic treatment did not improve antioxidant defense against challenge-induced oxidative stress. These mixed findings indicate that postbiotic efficacy on gut health is neither uniform nor universally positive.

### 6.3. Immune Modulation and Disease Resistance

Postbiotic and combined probiotic–postbiotic supplementation has been associated with improved immune-organ development, intestinal morphology, and gut microbial composition in broiler chickens [79]. Bastamy et al. [114] reported that a microbial-derived lysozyme preparation improved phagocyte activity and cytokine response in broiler chickens, illustrating the immune-enhancing potential of purified microbial components alongside whole-cell postbiotic preparations. This immune-enhancing capacity of postbiotics is especially advantageous in antibiotic-free poultry farming, where keeping flocks healthy and controlling disease outbreaks without antimicrobial use is critical. Effects on cellular immune parameters have not been consistent across all measures. Soren et al. [57] reported that neutrophil and lymphocyte phagocytic activity were not significantly different among treatment groups by day 35, indicating that postbiotic-associated immune enhancement is not uniform.

### 6.4. Stress Mitigation in Poultry Production

Heat stress remains a significant challenge in poultry production, reducing feed intake and growth and increasing mortality with rising ambient temperatures. Dunisławska and Bełdowska [115] mentioned that postbiotic supplementation may mitigate heat stress in poultry via modulation of the gut–brain axis, potentially through SCFAs and GABA, acting at the level of the hypothalamic-pituitary-adrenal (HPA) axis to attenuate corticosterone release. Consistent with this, postbiotic supplementation has been directly associated with reduced circulating corticosterone and heterophil-to-lymphocyte ratio in heat-challenged broilers [93], alongside improved feed conversion under stress conditions. Roque et al. [63] similarly synthesized evidence that postbiotics can reduce corticosterone levels and support water retention and electrolyte balance during heat stress, reinforcing their potential role in physiological stress mitigation. Not all parameters improved alongside these physiological markers. In a heat stress trial by Price et al. [80], body weight and flock uniformity did not differ significantly between treatments and plasma heat-shock protein 70 (HSP70) concentrations were unaffected by the supplementation, rising only in response to heat stress itself regardless of dietary treatment. This suggests postbiotic-mediated amelioration of heat stress may act preferentially on glucocorticoid and cellular immune indices rather than uniformly across all cellular stress biomarkers.

### 6.5. Oxidative Stress and Antioxidant Activity

Oxidative stress arises when reactive oxygen species production exceeds the host’s antioxidant capacity, leading to cellular damage and impaired physiological functions, a well-recognized consequence of common commercial stressors in poultry production [116]. Postbiotic components counter oxidative stress through two distinct routes: direct free-radical scavenging by certain metabolites and indirect potentiation of host antioxidant enzyme systems [29]. Girija et al. [72] reported that postbiotic supplementation increased the activity of glutathione peroxidase (GPx) and superoxide dismutase (SOD) levels, while improving overall antioxidant status and reducing lipid peroxidation in poultry meat, confirming an indirect effect of postbiotics through potentiation of the host’s physiological state. The direct role of postbiotic components in scavenging free radicals has been demonstrated in a number of studies employing in vitro assays like DPPH, OH, and ABTS scavenging activities [117,118]. The antioxidant response is not uniform across all enzyme systems or postbiotic sources. In a heat-stress trial by Humam et al. [45], SOD and GPx activities were not significantly affected, in contrast to the GPx/SOD increases reported by Girija et al. [72]. This suggests that the specific antioxidant enzymes potentiated by postbiotic supplementation may be strain- and product-dependent rather than uniform across the antioxidant enzyme system.

### 6.6. Product Quality and Nutritional Value

Consumer acceptance of poultry products depends on the quality of meat and eggs, which ultimately affects market value. Similarly, antioxidant capacity, plasma lipid profile (including cholesterol levels), and meat texture influence both the nutritional quality and commercial value of poultry products. Several studies have highlighted the positive impact of postbiotics on poultry product quality. Loh et al. [51] reported that postbiotic supplementation reduced plasma and yolk cholesterol levels while enhancing antioxidant capacity and reducing lipid oxidation [45], which contributed to improved egg quality and extended shelf life of broiler meat. In a comparative evaluation of probiotic, postbiotic, and prebiotic supplementation, Mohammed and Kareem [62] mentioned improved meat tenderness and water-holding capacity in postbiotic-supplemented broilers, alongside comparable benefits in the other treatment groups. These enhancements not only promote consumer health but also increase the commercial value of poultry products, underscoring the potential of postbiotics as a functional additive in poultry production. However, not all yeast-product trials report benefit. In a short-term feeding study, Korver et al. [94] found that commercial yeast fermentation products had little effect on egg production or shell quality in laying hens, and one product (Hilyses) was associated with reduced shell weight and thickness relative to control, underscoring that product- and strain-specific composition determines outcomes rather than yeast-derived postbiotics acting as a uniform class.

## 7. Routes of Postbiotic Administration in Poultry

Postbiotics, alone or in combination with other bioactive compounds, have been administered through oral (feed and/or water) to birds and in ovo embryos (Figure 3). Table 2 and Table 3 summarize study outcomes by route and distinguish postbiotic-only interventions from combination treatments where applicable.

**Table 3:** Effects of postbiotics in combination with probiotics, prebiotics, or phytogens administered through feed in chickens: An overview.

Sr. No.	Producer Strain	ISAPP Cat	Postbiotics (IM)	In Combination	Route	Duration	Bird Type	Sample Size	Comparators	Effects	Ref
1	*L. plantarum* RG14 and RI11	(iv)	CFS (Centrifuge)	Inulin (prebiotic)	Feed	42 days	Broilers	Day-old 300 Broiler chicks, divided into diff groups	NC (negative control), PC (positive control, antibiotic), T1–T4 (RG14 postbiotic ± inulin combinations)	Improved growth performance and FCR; red Enterobacteria and *E. coli*; upregulated IL-6	[112]
2	*L. plantarum*	(iv)	CFS (Centrifuge)	Phytogenics	Feed	35 days	Broilers	288 one-day-old unsexed Ross 308 chicks, 6 treatments × 4 replicates × 12 birds	N-control; P-control (diet + antibiotic); four postbiotic + thyme oil dose tiers (0.05/0.05% up to 0.2/0.2%)	Improved growth performance, higher BWG, lower FCR, increased antibody titers	[119]
3	*L. plantarum* RG14, *L. plantarum* RI11	(iv)	liquid metabolite postbiotic (10,000× *g*, 15 min)	Inulin (prebiotic)	Feed	42 days	Broiler	288 one-day-old chicks, 8 treatment groups, 6 replicates × 6 birds	NC, PC + neomycin + oxytetracycline); T1 (0.3% RI11 alone); T2 (0.3% RG14 alone); T3–T6 (RI11 or RG14 combined with 0.8% or 1.0% inulin)	Improved body weight and FCR; increased LAB and VFA; reduced *Enterobacteriaceae* and pH; enhanced liver IGF1 and GHR expression	[44]
4	*L. plantarum* RG14 and RI11	(iv)	Fermentation	Inulin (prebiotic)	Feed	42 days	Broilers	300 day-old birds	Control vs. Pre and postbiotics	Reduced drip loss, improved breast meat lightness	[120]
5	*S. cerevisiae* and phytase co-fermentation of wheat bran	(iii)	Fermented wheat bran (*S. cerevisiae* + phytase co-fermentation)	Fermentation-based (10% FWB tested)	Feed	35 days	Broilers	300 male, Ross 308, 5 groups, 3 reps	Control vs. 4 fermentation treatments	Growth, antioxidation, immunity, intestinal morphology effects	[78]

### 7.1. Administration of Postbiotics Through Feed

Postbiotics, alone or in combination with other compounds, have most commonly been incorporated into the diet via spraying, valued for its ease of use, stability, and cost-effectiveness (Table 2 and Table 3). Growth performance was the most frequently reported outcome [43,86,87,91,92]. Improvements in gut health, including enhanced intestinal morphology and microbial balance, were reported by Guan et al. [84] and Sharma and Kim [121]. Immune modulation, reflected by increased antibody levels and cytokine regulation, was reported in several studies, including a postbiotic-saponin combination product evaluated with and without concurrent vaccination against colibacillosis [89]. Additionally, several studies addressed pathogen control [88,91], oxidative stress reduction [90], and egg production and quality [51]. Under heat-stress conditions specifically, *L. plantarum*-derived postbiotics improved growth performance, carcass yield, intestinal morphology, and immune status in broilers [43] and supported layer hen performance [91]. A *Bacillus subtilis*-derived postbiotic improved growth performance, immunity, and tibia health while reducing mortality in broiler chicks in a dose-dependent manner, with the strongest effect at 0.015% inclusion [92]. These findings underscore the broad functional potential of postbiotics in poultry production.

### 7.2. Administration of Postbiotics Through Drinking Water

Administering postbiotics via drinking water may offer advantages over feed supplementation, particularly under conditions where feed intake is reduced (e.g., disease, heat stress). This enables uniform and rapid distribution of bioactive compounds present in postbiotic preparations. Studies have reported that postbiotics given in drinking water improve gut morphology (villus height and VH:CD) and tight junction gene expression and reduce inflammatory responses in broiler chickens during intestinal challenge [82,121] (Table 2). A postbiotic–phytobiotic combination administered via drinking water similarly contributed to improved growth performance and immune response [119]. A postbiotic administered both in ovo and via drinking water was associated with improved average daily gain and reduced intestinal lesion scores during a subclinical necrotic enteritis challenge [122]. (This study is discussed here as well as in Section 7.3, since it used a dual-route design rather than water delivery alone). Although fewer studies have explored water-based delivery compared to feed supplementation, interest in this route is growing due to its ease and even distribution of postbiotic components. However, current studies vary in terms of dosage, duration, and formulation, highlighting the need for further research to establish standardized protocols and to assess long-term efficacy across all delivery methods.

### 7.3. Administration of Postbiotics Through In Ovo Delivery

In ovo administration represents an innovative approach in poultry production, delivering bioactive compounds directly to the embryos [123,124]. This strategy is proposed to facilitate early modulation of gut microbiota and support immune competence along with intestinal development. A postbiotic delivered in ovo, combined with post-hatch drinking water supplementation, was associated with improved average daily gain, reduced jejunal and ileal lesion scores, and upregulated nutrient transporter gene expression (SGLT1, GLUT2, EAAT3) during a subclinical necrotic enteritis challenge [122]. Synbiotics (postbiotic–prebiotic combination) delivered in ovo have been associated with altered small intestinal microstructure at hatch and at 42 days [125]. Overall, research on in ovo postbiotic delivery remains limited, and current findings should be regarded as a basis for future hypothesis-driven research rather than an established production strategy. Whether early gut and immune modulation translates into reduced reliance on post-hatch antibiotic use has not yet been established. Further studies are also required to optimize in ovo dosage, timing, safety, and formulation consistency across different poultry species and production systems.

In ovo delivery of a *S. cerevisiae*-derived postbiotic, combined with post-hatch water supplementation, significantly upregulated intestinal nutrient transporter genes SGLT1, GLUT2, and EAAT3 in broilers, alongside improved ADG and reduced NE lesion scores during subclinical necrotic enteritis challenge [122]. A companion study using the same experimental cohort further showed that this postbiotic regimen improved intestinal permeability, histomorphology, and immune-related gene expression under NE challenge, indicating benefits extending beyond nutrient transport alone [61]. For context, earlier work has shown that in ovo delivery of prebiotic carbohydrates can also prime nutrient transporter expression ahead of hatch. Mannan-oligosaccharide increased SGLT1 and PepT1 mRNA abundance and enterocyte maturity at hatch [126] and in ovo galacto-oligosaccharide injection similarly elevated sodium-dependent glucose co-transporter levels linked to monosaccharide absorption. However, galacto-oligosaccharides are prebiotics, not postbiotics, interventions, and are noted here only to show that in ovo nutrient-transporter priming is a broader phenomenon across bioactive feed compounds. They should not be read as evidence of postbiotic efficacy specifically. Evidence for in ovo postbiotic delivery itself remains limited to the single study described above [60,122], and conclusions about its effectiveness should be correspondingly cautious.

### 7.4. Post-Harvest and Food Safety Applications

Beyond in-production administration, postbiotic preparations have also been evaluated as a post-harvest food-safety intervention. Reddyvari and Amalaradjou [127] reported that postbiotic wash treatments reduced *Salmonella* contamination on and within eggs while maintaining egg quality, representing a distinct application context from the in-production interventions discussed above. Similarly, Serter et al. [128] found that postbiotics derived from *P. acidilactici*, *L. plantarum*, and *Latilactobacillus sakei* extended the shelf life of chicken breast meat by 8–9 days beyond the 5-day shelf life of untreated controls, alongside reductions in *Salmonella* spp. and *L. monocytogenes* of 0.9–1.8 log_10_ CFU/g.

## 8. Role of Postbiotics in One Health Perspective

Incomplete implementation of the NAP-AMR across Pakistan’s agricultural and food sectors has perpetuated heavy reliance on antibiotics in commercial poultry production [129]. The resulting antibiotic residues in poultry products constitute a significant barrier to international meat exports and pose a serious One Health hazard at the interface of human, animal, and environmental health [130]. Within this context, postbiotics have emerged as a viable functional feed strategy for reducing reliance on prophylactic and therapeutic antimicrobials. Postbiotic supplementation has been proposed as a promising path for mitigating AMR and reducing antibiotic residue risks across poultry value chains. Dietary inclusion of SCFP is associated with significantly lower *Salmonella* cecal prevalence and litter load in broiler chickens under commercial farm conditions [59] and postbiotic-associated metabolites have been shown to reduce *Campylobacter* cecal colonization in broilers in an in vitro broiler cecal culture model [130], providing direct primary-study support for postbiotic suppression of these two zoonotic pathogens in poultry. Evidence for postbiotic suppression of extended-spectrum β-lactamase (ESBL)-producing organisms in poultry remains more limited and is currently supported primarily by review-level synthesis [23] rather than a dedicated primary study. This is a specific, narrower evidence gap and should not be implied to have the same level of direct support as the *Salmonella* and *Campylobacter* findings above. Despite promising outcomes, postbiotic pathogen suppression remains inconsistent across certain endpoints. For instance, Chaney et al. [59] reported that environmental litter prevalence did not differ significantly between treatments. Furthermore, Feye et al. [130] demonstrated that anti-*Campylobacter* efficacy was entirely contingent upon the resident cecal microbiota and the postbiotic alone failed to reduce pathogen recovery in isolation. This indicates that suppression is driven by microbiome-mediated competitive exclusion rather than the direct antimicrobial activity of the postbiotic metabolites.

## 9. Challenges and Future Directions

Although ISAPP has provided a widely cited concise definition for postbiotics [30], this effort emerged from a background of inconsistent usage of alternative terminology, historically applied to inactivated microbes, isolated metabolites, or fermentation products (Section 3), leading to methodological ambiguity and a lack of comparability across studies. While the definitional question itself is now substantially resolved, the current challenge is a lack of accurate and harmonized product characterization, production standards, and regulatory implementation across jurisdictions. Existing regulatory systems were designed around separate categories, such as feed additives, zootechnical additives, microbial preparations, and veterinary medicines, each with distinct approval pathways and evidentiary requirements. Postbiotics currently lack a dedicated regulatory category in most jurisdictions. Consequently, an individual product is evaluated based on its intended use and claims: as a feed additive for nutritional or sensory quality, as a zootechnical additive for growth performance or gut flora modulation, or as a veterinary medicine for disease protection. This case-by-case treatment, rather than a single harmonized category, is itself part of the regulatory ambiguity discussed here. The regulatory treatment of postbiotics varies by classification. In the EU, microbial feed products are typically authorized as zootechnical additives under Regulation (EC) No 1831/2003 of the European Parliament and of the Council of 22 September 2003 on additives for use in animal nutrition [131], requiring a full EFSA safety and efficacy dossier. In the United States, comparable substances were historically regulated as animal drugs, with the FDA Center for Veterinary Medicine withdrawing its 1998 Policy and Procedures Manual Guide 1240.3605 (Regulating Animal Foods with Drug Claims) on 20 May 2024 [132,133] and transitioning toward a new “zootechnical animal food substance” category, a process still under legislative development via the proposed Innovative Feed Act [134].

Reported efficacy is also not uniform across outcomes: intestinal permeability [95], final body weight [93], added growth benefit from combination with a live probiotic [79], and shell quality/egg production [94] were each unaffected or non-significant in at least one study despite improvements reported for other endpoints in the same or related trials, underscoring that postbiotic efficacy is frequently endpoint-specific rather than broadly and uniformly protective.

The main technical challenge is establishing analytical parameters covering minimum active metabolite concentration, producer-strain safety, absence of antibiotic resistance genes, and reproducibility. The genuine variation in strain, substrate, and inactivation method continues to complicate standardization, reproducibility, and safety evaluation across the field. Salminen et al. [30] emphasized the need for standardized production and characterization guidelines to improve reproducibility in postbiotic research. Future research should prioritize harmonized evaluation frameworks for the production, quality control, and efficacy testing of postbiotics. These frameworks would help to define critical parameters such as composition, stability, and dosage.

Secondly, a formal cost-benefit analysis for postbiotics in poultry production (particularly in low-resource settings) has not yet been conducted and represents an important gap for future research. Abd El-Ghany et al. [82] and many other researchers reported improved growth performance and gut health outcomes in postbiotic-supplemented broilers. Whether these benefits on performance can be translated into net economic benefit has not been formally assessed by this or other cited sources and remains an open question. Improved biological performance does not automatically translate into lower total production cost once product price, inclusion rate, feed processing and storage costs, and variability in biological response are accounted for.

Moreover, continued innovations in fermentation sustainability and the efficient use of raw materials will be needed to scale postbiotic production, reflecting the greater complexity of postbiotics relative to probiotics while suggesting greater opportunities for innovation. Repurposing residual microbial biomass from postbiotic production (for example as a soil amendment) has been proposed as a potential circular-economy application. Such reuse would require evaluations of microbial viability, strain identity, absence of AMR genes, contaminant and substrate-residue levels, ecotoxicity, and applicable regulatory approval before it could be considered safe practice. Reusing production-stage microbial cells directly as probiotics is inconsistent with the inactivation process central to postbiotic manufacture and would raise separate safety and regulatory questions specific to live-organism products. These circular-economy applications, if developed and validated responsibly, could contribute to the long-term sustainability of postbiotic production in poultry systems.

The integration of postbiotics with precision agriculture technologies represents an under-explored area for future research. Sensors and data analytics to monitor feed intake, water intake, body weight, environmental temperature, and disease indicators could, in principle, support real-time optimization of postbiotic dosing and evaluation of return on investment at the flock level. This remains a proposed direction for future research rather than an established application; no primary studies applying precision-agriculture frameworks specifically to postbiotic use in poultry were reported so far.

The current evidence supports the potential of postbiotics to improve the intestinal health, growth performance, immune system, and stress tolerance in poultry. However, the number of field studies remains limited, and further research is required to substantiate these benefits and clarify their underlying mechanisms in detail. Considerable scope remains for research on the modes of action of individual postbiotic components, indicating which metabolites or cellular structures are primarily responsible for specific effects at a given dose. In addition, dose–response, strain- and substrate-specific differences, and potential synergistic effects between components require systematic investigation. Postbiotics are produced under varying fermentation conditions (such as temperature, pH, oxygen availability, and nutrient sources) and through defined multi-strain systems. Since these parameters may alter microbial metabolism and consequently influence the composition and functionality of resulting bioactive metabolites, the future research priorities can be summarized as follows: (i) chemical and physical characterization of postbiotic preparations; (ii) batch-to-batch consistency testing; (iii) validated inactivation methods; (iv) dose–response studies in poultry; (v) long-term safety and residue/withdrawal evaluation; (vi) multisite commercial field trials; (vii) interaction effects with vaccination and concurrent medication; and (viii) the influence of host age, genetics, and baseline microbiota composition on postbiotic efficacy.

As mentioned in the Methodology (Section 2), the literature search for this review was limited to PubMed and Google Scholar, supplemented by citation screening. Scopus, Web of Science, CAB Abstracts, and Embase were not searched directly due to a lack of institutional subscription access. This constraint may have limited the comprehensiveness of the evidence base summarized here and should be addressed in future systematic or scoping reviews of this literature with broader database access.

## 10. Conclusions

Selected postbiotic preparations show promise as an alternative to antibiotic growth promoters in poultry production, with numerous experimental studies demonstrating beneficial effects on growth performance, gut health, and immune function. However, efficacy appears product- and strain-specific, and current evidence is not yet sufficient to support postbiotics as a universal AGP replacement. Key challenges include inconsistent findings across studies, the frequent use of mixed or complex products rather than well-characterized postbiotic-only preparations, limited commercial field validation, and gaps in harmonized production standards and regulatory frameworks. Addressing these knowledge gaps will require systematic in vivo trials with well-defined comparator groups, standardized production and characterization protocols, and comprehensive assessments of economic feasibility across diverse production systems. Coordinated, multidisciplinary research, spanning microbiology, nutrition, veterinary science, and legislation, will be needed to translate the promising findings summarized in this review into reliable, commercially viable applications.

## Figures and Tables

**Table 1:** Evolution of postbiotic terminology in literature and scope of the ISAPP consensus definition: a comparison.

Sr.#	Term	Definition	Within ISAPP Postbiotics Scope?	Refs
1	Paraprobiotic	Non-viable microbial cells (intact or broken), or crude cell extracts, which, when administered, confer a benefit. Term coined specifically for this purpose.	Yes, considered a subset of postbiotics emphasizing cellular components.	[31]
2	Tyndallized probiotic	Probiotic cells inactivated via tyndallization (fractional heat sterilization); used interchangeably with “ghost probiotic” and “paraprobiotic” in early literature.	Yes, as an inactivation method that can yield a postbiotic.	[28]
3	Ghost Probiotic	Same origin as “paraprobiotic”, non-viable microbial cells retaining structural integrity.	Yes, synonymous usage with paraprobiotic in the founding paper.	[31]
4	Metabiotics	Term used by some authors to collectively refer to microbial metabolites conferring a health benefit.	No, ISAPP explicitly excludes metabolite-only preparations lacking cell biomass.	[30]
5	Cell-free supernatant (CFS)	The soluble/liquid fraction secreted by live bacteria or released after lysis, containing enzymes, peptides, teichoic acids, organic acids, etc.	Ambiguous/generally no, if no cell biomass remains, ISAPP recommends the collective term “cell-free filtrate” rather than “postbiotic”. However, some literature like Nataraj et al. [32] claims that postbiotics naturally encompass CFS and filtrates, classifying them as structural horizons in biotherapy because the metabolic end products independently drive host health benefits.	[27,32]
6	Purified metabolites (e.g., isolated SCFA, bacteriocin)	A single, chemically defined compound derived from a microbial source.	No, ISAPP explicitly excludes purified metabolites in the absence of cell biomass; these should be named by their chemical identity instead (e.g., “butyric acid,” not “postbiotic”)	[30]
7	Bacterial lysate	Cell contents released by mechanical, chemical, or enzymatic lysis, retaining cellular components.	Yes, if cellular components remain in the preparation.	[27]
8	Biogenic/abiotic components	General umbrella term for non-living bioactive substances of microbial origin.	Ambiguous, depends on whether cellular structure is retained; overlaps with “postbiotic” only when it does.	[27]

## Data Availability

No new data were created or analyzed in this study. Data sharing is not applicable to this article.

## Abbreviations

The following abbreviations are used in this manuscript:

AGP	Antibiotic growth promoter
AMR	Antimicrobial resistance
EU	European Union
FAO	Food and Agriculture Organization of United Nations
SCFA	Short chain fatty acids
CFS	Cell-free supernatant
ISAPP	International Scientific Association of Probiotics and Prebiotics
LAB	Lactic acid bacteria
*L. plantarum*	*Lactiplantibacillus plantarum*
*S. cerevisiae*	*Saccharomyces cerevisiae*
SCFP	*Saccharomyces cerevisiae* fermentation product
FCR	Feed conversion ratio
LTA	Lipoteichoic acid
TA	Teichoic acid
EPS	Extracellular polysaccharides
GABA	Gamma-aminobutyric acid
DON	Deoxynivalenol
ZO-1	Zonula occludens-1
NF-κB	Nuclear factor kappa B
MAMPs	Microbe-associated molecular patterns
PRRs	Pattern recognition receptors
TLRs	Toll-like receptors
NOD	Nucleotide-binding oligomerization domain
IL-10	Interleukin-10
TNF-α	Tumor necrosis factor-alpha
EGFR	Epidermal growth factor receptor
IECs	Intestinal epithelial cells
FXR	Farnesoid X receptor
TGR5	Takeda G-protein-coupled receptor 5
GLP-1	Glucagon-like peptide-1
CNS	Central nervous system
*E. coli*	*Escherichia coli*
APEC	Avian pathogenic *Escherichia coli*
HPA	Hypothalamic-pituitary-adrenal
GPx	Glutathione peroxidase
SOD	Superoxide dismutase
MIP	Macrophage inflammatory protein
IFN	Interferon
FWB	Fermented wheat bran
ADG	Average daily gain
ADFI	Average daily feed intake
ESBL	Extended-spectrum beta-lactamase
VH:CD	Villus height to crypt depth ratio
NDV	Newcastle disease virus
IBDV	Infectious bursal disease virus
IBV	Infectious bronchitis virus
IgA	Immunoglobulin A
IgG	Immunoglobulin G
NLRP3	NLR family pyrin domain containing 3
SE	*Salmonella enterica* serovar Enteritidis
MUC	Mucin
HSP	Heat shock protein
α1-AGP	alpha-1-acid glycoprotein
SIgA	Secretory immunoglobulin A
VRE	Vancomycin-resistant *Enterococcus*
FI	Feed intake
NSP	Non-starch polysaccharides
LITAF	Lipopolysaccharide-induced tumor necrosis factor-alpha factor
VFA	Volatile fatty acids
NE	Necrotic enteritis
SGLT	Sodium-glucose linked transporters
GLUT	Glucose transporters
EAAT	Excitatory amino acid transporter
AMPK	AMP-activator protein kinase
PC	Positive control
NC	Negative control
